# Telepsychiatry implementation in Qatar: A three-year analysis of no-show rates and its impact on mental health service delivery

**DOI:** 10.5339/qmj.2024.50

**Published:** 2024-12-27

**Authors:** Ovais Wadoo, Faisal Khan, Javed Latoo, Yasser Saeed Khan, Prem Chandra, Yousaf Iqbal, Raed Amro, Shuja Reagu, Majid Alabdulla

**Affiliations:** ^1^Department of Psychiatry, Hamad Medical Corporation, Doha, Qatar; ^2^College of Medicine, Qatar University, Doha, Qatar; ^3^Medical Research Center, Hamad Medical Corporation, Doha, Qatar; ^4^Weill Cornell Medicine, Doha, Qatar *Correspondence: Ovais Wadoo. Email: owadoo@hamad.qa

**Keywords:** Telepsychiatry, telepsychology, no-show rates, care accessibility

## Abstract

**Background:** The COVID-19 pandemic triggered a rapid shift to telehealth and reshaped healthcare delivery worldwide. In Qatar, telehealth has emerged as a critical tool for ensuring uninterrupted care while minimizing the spread of viruses. However, its long-term efficacy, particularly in mental health services, is still poorly understood. The aim of this study was to address this gap by evaluating the post-pandemic impact of telehealth on the accessibility of mental health services in Qatar.

**Methods:** We conducted a comparative analysis of no-show rates over a four-month period before the initiation of telepsychiatry and telepsychology (March to June 2019) and corresponding periods in 2020, 2021, and 2022. The analysis considered visit types (new visit and follow-up visit) in various psychiatric (child and adolescent, adult, geriatric, forensic, and intellectual disability) and psychological outpatient clinics before and after the implementation of telehealth services.

**Results:** Among both new and follow-up cases, there was a significant reduction (*p* < 0.0001) in the collective proportion of no-show rates following the introduction of telehealth compared to the rates recorded before the implementation. Exceptions to this trend were appointments in child and adolescent mental health service psychology and forensic psychiatric services.

**Conclusion:** Telehealth has proven instrumental in improving access to mental health services in Qatar post-pandemic. Its integration shows the potential for reducing no-show rates and enhancing continuity of care. These findings are important for healthcare policy-making and practice in Qatar and provide valuable insights into the global discourse on the evolving role of telehealth.

## INTRODUCTION

Mental illness affects approximately 25% of the world's population annually. Despite this widespread prevalence, there is still a significant gap in treatment accessibility. Factors contributing to this treatment gap include underfunding, lack of trained staff, stigma, poor organization of care, and inefficient use of services.^1^ In resource-rich countries such as those in the Gulf Cooperation Council, gaps and delays in mental health treatment are influenced by factors beyond financial constraints. Despite significant investments in healthcare infrastructure, there may still be a shortage of specialist mental health professionals. Stigma surrounding mental illness remains a significant barrier and often discourages people from seeking help.^2^ Lack of awareness or education about mental health can delay recognition of symptoms and treatment. Cultural norms that prioritize seeking help from faith healers contribute to underutilization of services. In resource-poor countries, financial barriers prevent many from seeking medical care. Fragmented healthcare systems and long waiting times also contribute to delays.^3^ Information from research in 17 countries shows that the majority of individuals who developed a mental disorder within a 12-month period did not receive any treatment, especially in less developed countries.^4^ This situation is further complicated by significant delays in seeking treatment, as individuals must wait extended periods of time before receiving appropriate care. For example, individuals with anxiety disorders may endure delays of up to 30 years to seek treatment, while those with mood disorders may wait approximately 14 years, and individuals struggling with substance use disorders may delay treatment for up to 18 years.^5^ These findings highlight the urgent need for improved access to mental health services (MHS) and timely interventions, particularly in regions with limited resources and support systems. The repercussions of untreated mental health and substance use disorders are profound, resulting in both personal distress and significant economic burden.^6^ As healthcare organizations struggle with increasing demand and fiscal constraints, optimizing the effectiveness and efficiency of their services is essential. The burden of mental disorders, which ranks among the top 10 causes of disability-adjusted life years worldwide, emphasizes the urgent need for adequate resources and services to bridge this treatment gap.^7^ Urgent action is needed to address this issue and ensure equal access to mental healthcare for all individuals in need.

Patient non-attendance is a significant measurable outcome that highlights the mismatch between available resources and expected demand. These absences not only disrupt healthcare operations, but also squander valuable human and spatial resources, impacting service quality and performance. Patient non-attendance, commonly referred to as “no-shows” or “did not attend” in the medical context, remains a significant problem for healthcare providers across various clinical disciplines and settings.^8–10^ This phenomenon is characterized by patients not attending their appointments without prior notice of cancellation. Moreover, a long waiting time is associated with a higher rate of non-attendance in outpatient clinics.^11^ The reported rates of no-shows indicate significant variation, particularly within psychiatric outpatient clinics, ranging from 2% to 30%.^12^ Psychiatric clinics affiliated with academic medical institutions record significantly higher no-show rates, with initial psychiatric evaluations showing a twofold increase in non-attendance compared to other medical specialties.^13,14^ Community mental health clinics are also reported to experience some of the highest no-show rates and longer wait times for new evaluations compared to other healthcare subspecialties.^13^ Overall, no-show rates can be as high as 60% in mental health settings.^15,16^ For healthcare providers, the consequences of patient no-shows are multifaceted and include lost time, increased healthcare costs, reduced productivity and efficiency, and limited facility capacity.^17–19^ Patients, in turn, experience reduced satisfaction and compromised quality of care, as no-show incidents hinder their timely access to essential health services.^20,21^ The impact is particularly evident in mental health settings, where failure to connect individuals with mental disorders to services results in loss of follow-up, clinical deterioration, inappropriate emergency department use, and delayed treatment onset. Moreover, for patients recently discharged from inpatient units, non-attendance at initial outpatient appointments is predictive of higher rehospitalization rates and adverse outcomes.^4,22^ Beyond individual repercussions, the societal burden of no-shows extends to delayed treatment for other patients waiting for a consultation, contributing to inefficient use of staff time and resource wastage.^23^

Telepsychiatry has emerged as a cost-effective solution for enhancing access to psychiatric care, supported by evidence demonstrating its feasibility, user acceptability, and effectiveness in improving outcomes and quality of life across various mental disorders.^24^ Studies indicate its equivalence to in-person care in terms of therapeutic alliance and patient satisfaction. Before telepsychiatry, various strategies were used to improve access to MHS. The aim of community care was to decentralize services by integrating them into primary healthcare. Task-shifting has allowed non-specialist healthcare workers to provide basic mental health support, thereby addressing the shortage of professionals. Public awareness campaigns focused on reducing stigma and encouraging help-seeking behavior. These efforts have often been limited by resource constraints. Particularly during the COVID-19 pandemic, the implementation of telepsychiatry has been associated with increased access and reduced no-show rates, fundamentally reshaping the landscape of mental healthcare delivery. However, current research primarily focuses on short-term periods before and after the adoption of telehealth services, with a dearth of follow-up studies exploring the sustained benefits of telehealth services.

In line with global trends, the State of Qatar swiftly embraced telehealth services in March 2020 amid the global pandemic.^25^ Hamad Medical Corporation, the main provider of government-funded healthcare services, including MHS in Qatar, witnessed widespread adoption of telehealth across various service levels, including community and outpatient services. These interventions not only addressed the immediate challenges of the pandemic, but also accelerated the advancement of telepsychiatry, promoting the transition from traditional in-person consultations to telehealth encounters. By leveraging technology and innovative delivery methods, MHS were able to adapt quickly and sustainably to the unprecedented demands of the COVID-19 crisis. A previous study in Qatar analyzed data on new and follow-up cases, as well as the rates of non-attendance to mental health outpatient settings in the first four months after the introduction of telepsychiatry, and showed a significant decrease in no-shows compared to the corresponding period before the implementation of telepsychiatry.^26^ Government-funded primary care centers and hospitals provide healthcare services to both citizens and migrants in Qatar.^27,28^ Numerous initiatives and legislative changes have been implemented over time to enhance access to care.^29–34^ While Qatar has a growing healthcare infrastructure, there is still a shortage of specialist mental health professionals and a need for more integrated primary care services. Public awareness campaigns are ongoing, but gaps in education and understanding of mental health issues remain, further delaying access to timely care. The aim of this present study was to explore the sustained impact of telehealth modalities on the MHS of Qatar in the subsequent three-year period following their introduction. By examining long-term trends and outcomes, this research aims to provide the first outcome data-based understanding of how telepsychiatry and telepsychology modalities continue to shape and optimize mental health service delivery in the Qatari context. The findings are expected to provide valuable insights for healthcare policy-makers, practitioners, and stakeholders navigating the evolving landscape of mental healthcare.

## METHODS

This was a retrospective observational study using administrative data from a single referral and booking system to analyze no-show rates. This method was faster and more cost-effective as it allowed us to use existing data to analyze large datasets and identify trends over time. However, this method has limitations in controlling for confounding factors such as patient demographics, clinical history, socioeconomic factors, or other external events. Data were analyzed by comparing no-show rates in the four months before the initiation of telehealth (March to June 2019) (pre-initiation period) with the corresponding periods in 2020 (post-initiation period 1), 2021 (post-initiation period 2), and 2022 (post-initiation period 3). The focus areas included child and adolescent mental health service (CAMHS) psychology, child and adolescent psychiatry, adult psychology, adult psychiatry, geriatric psychiatry, forensic psychiatry, and psychiatry of intellectual disability outpatient clinics. While this approach simplifies data collection, it can oversimplify complex phenomena and lead to the loss of important granular information. The aggregation of data into broad categories can obscure detailed patterns or variations within subspecialties, potentially affecting the depth and accuracy of the findings.

### Statistical analysis

Descriptive statistics were used to summarize data over periods before the initiation of telepsychiatry (year 2019) and after the implementation of the telepsychiatry program (2020, 2021, and 2022). The extended Mantel–Haenszel chi-square test was used to assess and statistically evaluate the overall linear trend in percentage of no-show rates in both new and follow-up cases. Furthermore, the chi-square test was used to assess statistically significant differences in no-show rates for both new and follow-up cases when comparing the periods before and after initiation across different subspecialties. The corresponding values of risk ratio (RR), absolute risk reduction (ARR), and 95% confidence interval (CI) were presented. All *p* values reported were two-tailed, and *p* values < 0.05 were considered statistically significant. All statistical analyses were performed using the statistical packages SPSS version 29.0 (IBM Corp., Armonk, NY) and Epi Info 2000 (Centers for Disease Control and Prevention, Atlanta, GA).

## RESULTS

**Figure 1.**
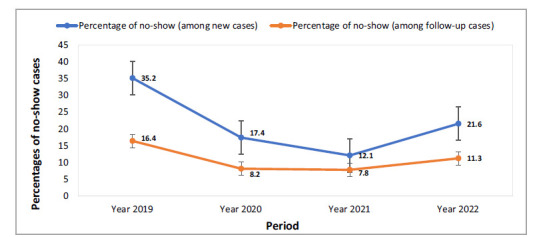
Percentages of no-show rates for new and follow-up cases between periods before the initiation of telepsychiatry (2019) and after the implementation of the telepsychiatry program (2020–2022). Extended Mantel–Haenszel chi-square for linear trend value: χ^2^ = 170.14, *p* < 0.0001 (for new cases) and χ^2^ = 184.6, *p* < 0.0001 (for follow-up cases). Error bars show 95% confidence intervals.

The collective proportion of no-show rates recorded a significant decrease (*p* < 0.0001) in both new and follow-up cases when comparing the rates before the introduction of the telehealth program (March-June 2019) with those during the corresponding months in the three years after its implementation (2020, 2021, and 2022) (Figure 1).

### Comparison of no-show rates for “new visits”

The overall percentage of no-show rates decreased significantly (from 35.2% to 17.42%; RR 2.02, 95% CI 1.8, 2.2; ARR 17.76%, 95% CI 15.6, 19.9; *p* < 0.0001) among new cases when comparing the period before initiation (2019) and after initiation period 1 (2020) of the telehealth program. Similar trends (decreased no-show rates) were observed across different subspecialties (*p* < 0.05), except CAMHS psychology, in which the difference in non-show rates was statistically insignificant (*p* = 0.930) (Table 1).

Statistical analysis showed that the overall percentage of no-show rates decreased significantly (from 35.2% to 12.11%; RR 2.91, 95% CI 2.6, 3.2; ARR 23.1%, 95% CI 21.0, 25.1; *p* < 0.0001) among new cases when comparing the period before initiation (2019) and after initiation period 2 (2021) of the telehealth program. Similar trends (decreased no-show rates) were observed across all subspecialties (*p* < 0.05), except CAMHS psychology, in which the difference in non-show rates was insignificant (*p* = 0.070) (Table 2 ).

Similar to the findings observed when comparing no-show rates between the pre-initiation (2019) and post-initiation periods 2 and 3 (2020 and 2021) of the telehealth program, the findings presented in Table 3 clearly indicate that the overall percentage of no-show rates also decreased significantly (from 35.2% to 21.6%; RR 1.63, 95% CI 1.5, 1.8; ARR 13.6%, 95% CI 11.4, 15.8; *p* < 0.0001) among new cases when comparing the period before initiation (2019) and after initiation period 3 (year 2022) of the telehealth program. Almost similar trends (decreased no-show rates) were observed across different subspecialties (*p* < 0.05). The differences in no-show rates were statistically insignificant for CAMHS psychology (*p* = 0.576) and forensic psychiatry (*p* = 0.087) clinics.

### Comparison of no-show rates for follow-up visits

Table 4 shows that the overall percentage of no-show rates decreased (from 16.42% to 8.15%; RR 2.01, 95% CI 1.9, 2.1; ARR 8.3%, 95% CI 7.6, 8.9; *p* < 0.0001) in the follow-up visits when comparing the period before initiation (2019) and after initiation period 1 (2020) of the telehealth program. Similar trends (decreased no-show rates) were observed across other subspecialties (*p* < 0.05), except CAMHS psychology (*p* = 0.498) and forensic psychiatry (0.1502).

The overall percentage of no-show rates decreased (from 16.42% to 8.15%; RR 2.01, 95% CI 1.9, 2.1; ARR 8.3%, 95% CI 7.6, 8.9; *p* < 0.0001) in the follow-up visits when comparing the period before initiation (2019) and after initiation period 2 (2021) of the telehealth program. Similar trends (decreased no-show rates) were also observed across other subspecialties (*p* < 0.05), except CAMHS psychology (*p* = 0.498) and forensic psychiatry (*p* = 0.150) (Table 5).

Similar to the findings observed when comparing no-show rates for the follow-up visits between the pre-initiation period (2019) and post-initiation periods 2 and 3 (2020 and 2021) of the telehealth program, the findings presented in Table 6 clearly indicate that the overall percentage of no-show rates also decreased significantly (from 16.42% to 11.25%; RR 1.46, 95% CI 1.4, 1.5; ARR 5.2%, 95% CI 4.5, 5.9; *p* < 0.0001) for the follow-up visits when comparing the period before initiation (2019) and after initiation period 3 (2022) of the telehealth program. Almost similar trends (decreased no-show rates) were observed across different subspecialties (*p* < 0.05). However, the differences in no-show rates in CAMHS (*p* = 0.145), CAMHS psychology (*p* = 0.612), and forensic psychiatry (*p* = 0.712) outpatient clinics were statistically insignificant.

## DISCUSSION

To our knowledge, this is the first study of its kind from the Middle East and North Africa region to analyze “no-show” rates and demonstrate the long-term benefits of telehealth services on accessibility of care and resource utilization in MHS. Although telehealth was started as a mitigation strategy to minimize the spread of infection during COVID-19, its long-term positive impact on access to healthcare has continued beyond the pandemic. The comparison of no-show rates in psychiatric and psychological clinics since the introduction of telehealth services is also unique to this study. Before the COVID-19 pandemic, the field of telepsychiatry in Arab countries, including Qatar, encountered numerous obstacles.^35^ These challenges included the reluctance of professionals to adopt telepsychiatry technologies. However, the outbreak of the COVID-19 crisis was an opportune moment for the evolution of telepsychiatry. During this period, healthcare providers experienced a notable learning curve and increased comfort in using telepsychiatry, paralleled by a notable acceptance of this modality among patients. Moreover, strategic policy adjustments were instrumental to the integration of telepsychiatry into clinical practice, contributing to a reduction in clinic no-show rates.

This study found a significant decrease in the no-show rates for both new and follow-up psychiatric and psychology visits during the study period compared to the rates recorded before the implementation of telehealth. Successful integration of telehealth depends on comprehensive education of patients and providers in technology use, robust staff and technical support systems, and implementation of policies that promote seamless access and widespread adoption of telehealth services.

Notable differences were observed in the reduction of no-show rates between different service types, with a statistically significant decrease observed in child and adolescent psychiatry but not in child and adolescent psychology outpatient clinics. Adolescents, deeply entrenched in online and digital spaces for social interaction, entertainment, self-expression, and education, seem to have found a natural fit in accessing telehealth services. However, the differential reduction in no-show rates between CAMHS psychology clinics and other clinics, particularly child and adolescent psychiatry clinics, demands careful examination within the context of telehealth use. Several factors may contribute to this finding. There may be a preference for psychiatric appointments, which could reflect patients' general tendency to trust medication interventions more than therapy. Additionally, families may view psychiatric appointments as essential for risk assessments, a service provided by psychiatrists. The perception that psychiatric sessions require less effort and time compared to psychology sessions may also influence this preference. Moreover, the scheduling and frequency of appointments could pose a challenge for psychology sessions, especially for children and adolescents who must balance academic demands. Psychological interventions typically require more frequent sessions than psychiatric consultations due to the nature of the work. School and other educational commitments can make it more difficult for patients to regularly attend psychology appointments, contributing to sustained no-show rates despite the availability of telehealth services. The lower attendance rates in psychology clinics could also be explained by the importance of direct human interaction. Therapy often relies heavily on the therapeutic alliance between the therapist and the patient, which is facilitated by in-person interactions. The absence of this direct human connection in telepsychology sessions can reduce patients' motivation to attend appointments.

Furthermore, the multifaceted nature of assessments and interventions in CAMHS psychology, which includes individual therapy, family-based interventions, school liaison, and collaboration with social services, may contribute to the disparity in findings.^36^ Similar observations were reported by Hoffnung et al., emphasizing the importance of tailoring telehealth engagement strategies to minimize information repetition and appointment confusion while maximizing care and comfort for children and their families.^37^ Over the past five decades, pediatric telepsychiatry practice has evolved significantly, with applications spanning developmental stages and various diagnostic categories.^38^ Developmental considerations and parental preferences should be taken into account when deciding the suitability of telepsychiatry interventions. To furnish clinicians with a clinical and evidence-based framework, the AACAP (American Academy of Child and Adolescent Psychiatry) has developed guidelines tailored to individuals interested in or actively involved in various levels of telepsychiatry implementation.^39^ These guidelines serve as a valuable resource for navigating the complexity of telepsychiatry practice and ensuring high-quality, evidence-based care.

In our forensic setting, we observed variable change in no-show rates despite the growing body of literature exploring the forensic applications of telepsychiatry. However, there is a noticeable dearth of research on the use of telepsychiatry in forensic settings in the Arab world. The most robust evidence for the use of telepsychiatry in forensic settings comes from countries characterized by expansive geographical areas and well-established forensic services that are seamlessly integrated into the criminal justice system, and often have virtual court systems.^40^ Studies conducted in secure hospitals and correctional facilities have demonstrated the safety and efficacy of telepsychiatry in conducting clinical assessments, providing benefits such as cost reduction, time savings, and minimized travel requirements. Moreover, the strategic allocation of specialist resources becomes more efficient through the implementation of telepsychiatry. Security aspects are of utmost importance in forensic MHS. Transporting patients outside of secure settings poses institutional risks, including the possibility of escape. However, the variable impact of telepsychiatry in our study setting can be attributed to the specific characteristics and infrastructure of forensic services in the region.^41^ Unlike contexts where secure hospitals and extensive integration into the criminal justice system are prevalent, our setting lacks such facilities. Virtual courtrooms are non-existent, and geographical distances pose minimal challenges given the country's compact size. Additionally, professionals in our setting lack formal clinical or legal guidelines and training opportunities specific to telepsychiatry in forensic contexts.^42^ These factors collectively contribute to the limited adoption and impact of telepsychiatry in our forensic setting.

Telepsychiatry has been shown to influence no-show rates. Offering remote consultations reduces barriers such as travel time and logistical challenges. The convenience of virtual visits and flexible scheduling options often aligns with patient preferences, increasing adherence and engagement. Telepsychiatry requires reliable technology and its users' expertise. Some patients may have technical difficulties or may not prefer virtual platforms. The digital divide refers to the gap between individuals with and those without access to digital technology and results from difficulties with technology or inadequate resources. This disparity can affect access, particularly impacting older adults, individuals with sensory impairments, those who are not tech-savvy, and marginalized groups. Patient preferences and insights play a significant role in influencing no-show rates in mental healthcare. It is crucial to understand the patient's preferences such as preferred modes of communication. Addressing issues such as digital literacy and providing support to those less comfortable with technology can further enhance engagement, ensure equitable access, and maximize the benefits of telepsychiatry for all populations.^43^

## LIMITATIONS

This study did not explore the specific reasons for clinic no-shows, as data collection was limited to aggregate no-show rates for each mode of care delivery. Individual-level data necessary for identifying changes in clinical symptoms and longitudinal outcomes were not available. Consequently, there was no way to determine whether the decrease in no-show rates following the transition to telehealth was associated with improved patient outcomes. The design of the study prevented a deeper understanding of the factors underlying the observed trends and their implications for patient care and treatment effectiveness.

## CONCLUSION

In summary, the implementation of telehealth has shown sustained improvements in reducing no-show rates. Despite its advantages, telehealth has several drawbacks. Technological barriers, including the need for reliable internet and devices, can exclude individuals with limited resources or poor digital literacy. Privacy concerns arise due to potential risks to patient confidentiality and data security. Additionally, telemedicine limits physical examination capabilities, which can impact the diagnosis and treatment of conditions that require in-person assessment. Reduced face-to-face interaction can affect the quality of the therapeutic relationship and communication. Regulations and reimbursement policies across regions can complicate the implementation and sustainability of telehealth services.

In order to integrate telepsychiatry into existing healthcare workflows, it is recommended to develop policies that promote equitable access and protect patient privacy. Future research investigating the determinants of clinic no-shows across different modalities of care delivery is essential to fully understand the factors influencing the no-show rates in virtual care settings. Prospective studies focused on elucidating whether the observed decrease in no-show rates can indeed translate into enhanced patient outcomes with telehealth interventions are crucial for informing evidence-based practices. Furthermore, there is an urgent need for future studies to assess the impact of demographic and socioeconomic factors on telehealth use. Understanding how these variables interact with telehealth use can inform strategies to mitigate disparities in access and engagement to promote equitable healthcare delivery. By addressing these research gaps, we can improve our understanding of the effectiveness and equity of telehealth services, optimizing patient care and outcomes in mental health settings.

Improving access to mental healthcare beyond telehealth requires several strategies. Expanding community-based services integrates mental healthcare into primary healthcare settings, making it more accessible. Task-shifting trains non-specialist professionals to provide basic mental health support, in order to address workforce shortages. Raising public awareness through educational campaigns helps reduce stigma and encourages early help-seeking. Enhancing insurance coverage and reducing financial barriers can also improve access. Finally, improving infrastructure and increasing the number of mental health professionals are crucial for expanding access to healthcare.

### Ethical considerations

Permission to publish these anonymized aggregate data was granted by hospital directors of the corresponding MHS. Patient records were not accessed and therefore IRB approval was not required.

### Authors' contributions

All authors contributed to the conception, analysis, and drafting of this manuscript.

### Competing interests

The authors have no conflicts of interest to declare.

## Figures and Tables

**Table 1.** Statistical comparison of the percentage of no-show (among new cases) across various subspecialties between 2019 and 2020.

**Subspecialties**	**No show (*n*/*N* (%)) (2019)**	**No show (*n*/*N* (%)) (2020)**	**RR (95% CI)**	**ARR (95% CI)**	**Chi-square (χ^2^) value**	***p***

Adult	379/1,307 (29%)	286/1,697 (16.9%)	1.72 (1.5, 2.0)	12.1% (9.1, 15.2)	63.18	< 0.0001

Allied health	62/164 (37.8%)	15/146 (10.3%)	3.68 (2.2, 6.2)	27.5% (18.6, 36.4)	31.36	< 0.0001

Psychology	383/872 (44%)	169/936 (18%)	2.43 (2.1, 2.84)	25.9% (21.8, 30)	142.4	< 0.0001

CAMHS	72/199 (36%)	40/164 (24%)	1.48 (1.1, 2.1)	11.8% (2.4, 21.2)	5.86	0.016

CAMHS psychology	58/156 (37.2%)	58/154 (37.7%)	0.98 (0.7, 1.3)	-0.48% (-11.3, 10.3)	0.08	0.930

LD	8/21 (38%)	1/47 (2%)	17.9 (2.4, 134.2)	35.97% (14.8, 57.1)	16.35	< 0.0001

Older adult	21/80 (26%)	7/151 (5%)	5.66 (2.5, 12.8)	21.61% (11.4, 31.8)	22.94	< 0.0001

Forensic	4/7 (57%)	2/27 (7%)	7.71 (1.8, 33.9)	49.74% (11.8, 87.7)	9.462	0.002

Total	987/2,806 (35.17%)	583/3,347 (17.42%)	2.02 (1.8, 2.2)	17.76% (15.6, 19.9)	253.2	< 0.0001


RR: risk ratio, CI: confidence interval, ARR: absolute risk reduction, LD: learning disability.

**Table 2.** Statistical comparison of the percentage of no-show (among new cases) across various subspecialties between 2019 and 2021.

**Subspecialties**	**No show (*n*/*N* (%)) (2019)**	**No show (*n*/*N* (%)) (2021)**	**RR (95% CI)**	**ARR (95% CI)**	**Chi-square (χ^2^) value**	***p***

Adult	379/1,307 (29%)	184/1,811 (10%)	2.85 (2.4, 3.4)	18.84% (16.01, 21.7)	182.1	< 0.0001

Allied health	62/164 (38%)	14/220 (6%)	5.94 (3.5, 10.2)	31.44% (23.4, 39.5)	58.51	< 0.0001

Psychology	383/872 (44%)	175/1,418 (12%)	3.56 (3.0, 4.2)	31.58% (27.9, 35.3)	292.2	< 0.0001

CAMHS	72/199 (36%)	35/206 (17%)	2.13 (1.5, 3.0)	19.19% (10.8, 27.6)	19.18	< 0.0001

CAMHS psychology	58/156 (37%)	72/252 (29%)	1.30 (1.0, 1.7)	8.61% (-0.81, 18.0)	3.289	0.0698

LD	8/21 (38%)	0/75 (0%)	–	38.1% (17.33, 58.86)	31.17	< 0.0001

Older adult	21/80 (26%)	26/236 (11%)	2.38 (1.42, 3.99)	15.23% (4.80, 25.7)	10.95	0.0009

Forensic	4/7 (57%)	2/53 (4%)	15.14 (3.4, 68.1)	53.37% (16.1, 90.4)	19.57	< 0.0001

Total	987/2,806 (35.17%)	508/4,196 (12.11%)	2.91 (2.64, 3.20)	23.1% (21.04, 25.09)	532.8	< 0.0001


RR: risk ratio, CI: confidence interval, ARR: absolute risk reduction, LD: learning disability.

**Table 3.** Statistical comparison of the percentage of no-show (among new cases) across various subspecialties between 2019 and 2022.

**Subspecialties**	**No show (*n*/*N* (%)) (2019)**	**No show (*n*/*N* (%)) (2022)**	**RR (95% CI)**	**ARR (95% CI)**	**Chi-square (χ^2^) value**	***p***

Adult	379/1,307 (29%)	333/1,797 (19%)	1.57 (1.4, 1.8)	10.47% (7.4, 13.5)	46.9	< 0.0001

Allied health	62/164 (38%)	78/500 (17%)	2.42 (1.8, 3.2)	22.2% (14.1, 30.3)	36.59	< 0.0001

Psychology	383/872 (44%)	330/1,137 (29%)	1.67 (1.5, 1.9)	17.54% (13.4, 21.7)	67.64	< 0.0001

CAMHS	72/199 (36%)	0/0 (%)	–	–	–	–

CAMHS psychology	58/156 (37%)	79/197 (40%)	0.93 (0.71, 1.21)	-2.92% (-13.14, 7.30)	0.313	0.576

LD	8/21 (38%)	4/59 (7%)	5.62 (1.9, 16.8)	31.32% (9.6, 53.1)	11.91	0.001

Older adult	21/80 (26%)	34/304 (11%)	2.35 (1.4, 3.8)	15.07% (4.8, 25.3)	11.71	0.001

Forensic	4/7 (57%)	2/11 (18%)	3.14 (0.8, 12.9)	38.96% (-4.20, 82.13)	2.92	0.087

Total	987/2,806 (35.17%)	860/4,005 (21.57%)	1.63 (1.51, 1.76)	13.6% (11.42, 15.78)	154.2	< 0.0001


RR: risk ratio, CI: confidence interval, ARR: absolute risk reduction, LD: learning disability.

**Table 4.** Statistical comparison of the percentage of no-show (among follow-up cases) across various subspecialties between 2019 and 2020.

**Subspecialties**	**No show (*n*/*N* (%)) (2019)**	**No show (*n*/*N* (%)) (2020)**	**RR (95% CI)**	**ARR (95% CI)**	**Chi-square (χ^2^) value**	***p***

Adult	1,618/10,429 (16%)	813/11,551 (7%)	2.2 (2.0, 2.4)	8.48% (7.6, 9.3)	400.3	< 0.0001

Allied health	272/2,352 (12%)	148/2,752 (5%)	1.64 (1.4, 1.9)	4.51% (3.1, 5.9)	55.25	< 0.0001

Psychology	398/1,596 (25%)	371/2,407 (15%)	1.62 (1.4, 1.8)	9.52% (7, 12)	56.09	< 0.0001

CAMHS	211/865 (24%)	130/868 (15%)	1.63 (1.34, 2.0)	9.42% (5.7, 13.1)	24.3	< 0.0001

CAMHS psychology	208/709 (29.3%)	190/686 (27.7%)	1.06 (0.9, 1.3)	1.64% (-3.1, 6.4)	0.46	0.498

LD	11/60 (18%)	5/267 (2%)	9.79 (3.53, 27.1)	16.46% (6.54, 26.4)	28.52	< 0.0001

Older adult	97/1,134 (9%)	32/2,191 (1%)	5.86 (4.0, 8.7)	7.09% (5.4, 8.8)	100.8	< 0.0001

Forensic	5/31 (16.1%)	25/300 (8.3%)	1.94 (0.8, 4.7)	7.80% (-5.5, 21.1)	2.072	0.1502

Total	2,820/17,176 (16.42%)	1,714/21,022 (8.15%)	2.01 (1.90, 2.13)	8.27% (7.60, 8.93)	617.3	< 0.0001


RR: risk ratio, CI: confidence interval, ARR: absolute risk reduction, LD: learning disability.

**Table 5.** Statistical comparison of the percentage of no-show (among follow-up cases) across various subspecialties between 2019 and 2021.

**Subspecialties**	**No show (*n*/*N* (%)) (2019)**	**No show (*n*/*N* (%)) (2021)**	**RR (95% CI)**	**ARR (95% CI)**	**Chi-square (χ^2^) value**	***p***

Adult	1,618/10,429 (16%)	953/12,476 (8%)	2.03 (1.9, 2.2)	7.88% (7.0, 8.7)	353.6	< 0.0001

Allied health	272/2,352 (12%)	161/3,001 (5%)	2.16 (1.8, 2.6)	6.2% (4.7, 7.7)	68.17	< 0.0001

Psychology	398/1,596 (25%)	260/2,564 (10%)	2.46 (2.1, 2.8)	14.8% (12.4, 17.2)	161.8	< 0.0001

CAMHS	211/865 (24%)	145/1,162 (12%)	1.96 (1.6, 2.4)	11.91% (8.5, 15.4)	48.62	< 0.0001

CAMHS psychology	208/709 (29%)	252/1,110 (23%)	1.29 (1.10, 1.51)	6.63% (2.5, 10.8)	10.08	0.0015

LD	11/60 (18%)	20/1,379 (1%)	12.64 (6.3, 25.2)	16.88% (7.1, 26.7)	77.75	< 0.0001

Older adult	97/1,134 (9%)	68/2,147 (3%)	2.70 (2.0, 3.7)	5.39% (3.6, 7.2)	45.08	< 0.0001

Forensic	5/31 (16.1%)	13/137 (9.5%)	1.7 (0.7, 4.4)	6.64% (-7.2, 20.5)	1.17	0.282

Total	2,820/17,176 (16.42%)	1,872/23,976 (7.81%)	2.10 (1.99, 2.22)	8.61% (7.96, 9.26)	734.5	< 0.0001


RR: risk ratio, CI: confidence interval, ARR: absolute risk reduction, LD: learning disability.

**Table 6.** Statistical comparison of the percentage of no-show (among follow-up cases) across various subspecialties between 2019 and 2022.

**Subspecialties**	**No show (*n*/*N* (%)) (2019)**	**No show (*n*/*N* (%)) (2022)**	**RR (95% CI)**	**ARR (95% CI)**	**Chi-square (χ^2^) value**	***p***

Adult	1,618/10,429 (16%)	1,249/12,287 (10%)	1.53 (1.4, 1.6)	5.35% (4.8, 6.2)	146.4	< 0.0001

Allied health	272/2,352 (12%)	303/3,570 (8%)	1.36 (1.2, 1.6)	3.08% (1.5, 4.7)	15.31	< 0.0001

Psychology	398/1,596 (25%)	550/2,588 (21%)	1.17 (1.0, 1.3)	3.69% (1.0, 6.3)	7.651	0.006

CAMHS	211/865 (24.4%)	266/1,227 (21.7%)	1.13 (1.0, 1.3)	2.71% (-1.0, 6.4)	2.123	0.145

CAMHS psychology	208/709 (29%)	201/657 (31%)	0.96 (0.8, 1.1)	-1.26% (-6.12, 3.61)	0.256	0.612

LD	11/60 (18%)	34/1,230 (3%)	6.63 (3.5, 12.4)	15.57% (5.7, 25.4)	41.19	< 0.0001

Older adult	97/1,134 (9%)	66/2,249 (3%)	2.92 (2.2, 4.0)	5.62% (3.8, 7.4)	51.9	< 0.0001

Forensic	5/31 (16.1%)	50/364 (13.7%)	1.17 (0.5, 2.7)	2.39% (-11.0, 15.8)	0.137	0.712

Total	2,820/17,176 (16.42%)	2,719/24,172 (11.25%)	1.46 (1.39, 1.53)	5.17% (4.49, 5.85)	231.3	< 0.0001


RR: risk ratio, CI: confidence interval, ARR: absolute risk reduction, LD: learning disability.
